# Nanomaterials Based on Collaboration with Multiple Partners: Zn_3_Nb_2_O_8_ Doped with Eu^3+^ and/or Amino Substituted Porphyrin Incorporated in Silica Matrices for the Discoloration of Methyl Red

**DOI:** 10.3390/ijms24108920

**Published:** 2023-05-17

**Authors:** Mihaela Birdeanu, Ion Fratilescu, Camelia Epuran, Liviu Mocanu, Catalin Ianasi, Anca Lascu, Eugenia Fagadar-Cosma

**Affiliations:** 1 National Institute for Research and Development in Electrochemistry and Condensed Matter, Plautius Andronescu Street 1, 300224 Timisoara, Romania; mocanuliv@gmail.com; 2 Institute of Chemistry “Coriolan Dragulescu”, Mihai Viteazu Ave. 24, 300223 Timisoara, Romania; ionfratilescu@acad-icht.tm.edu.ro (I.F.); ecamelia@acad-icht.tm.edu.ro (C.E.); ianasic@acad-icht.tm.edu.ro (C.I.); alascu@acad-icht.tm.edu.ro (A.L.)

**Keywords:** Zn_3_Ta_2_O_8_ oxides, Eu^3+^-doped mixed oxide, DFT calculations, porphyrin-Zn_3_Ta_2_O_8_-silica hybrids, methyl red adsorption

## Abstract

Designing appropriate materials destined for the removal of dyes from waste waters represents a great challenge for achieving a sustainable society. Three partnerships were set up to obtain novel adsorbents with tailored optoelectronic properties using silica matrices, Zn_3_Nb_2_O_8_ oxide doped with Eu^3+^, and a symmetrical amino-substituted porphyrin. The pseudo-binary oxide with the formula Zn_3_Nb_2_O_8_ was obtained by the solid-state method. The doping of Zn_3_Nb_2_O_8_ with Eu^3+^ ions was intended in order to amplify the optical properties of the mixed oxide that are highly influenced by the coordination environment of Eu^3+^ ions, as confirmed by density functional theory (DFT) calculations. The first proposed silica material, based solely on tetraethyl orthosilicate (TEOS) with high specific surface areas of 518–726 m^2^/g, offered better performance as an adsorbent than the second one, which also contained 3-aminopropyltrimethoxysilane (APTMOS). The contribution of amino-substituted porphyrin incorporated into silica matrices resides both in providing anchoring groups for the methyl red dye and in increasing the optical properties of the whole nanomaterial. Two different types of methyl red adsorption mechanisms can be reported: one based on surface absorbance and one based on the dye entering the pores of the adsorbents due to their open groove shape network.

## 1. Introduction

Recent research is increasingly focused on hybrid materials containing two or three partners belonging to different chemical classes that bring their best properties to the newly designed material. In this respect, researchers combined mixed oxides, porphyrins, and silica or polymeric materials [1] with the purpose of being used in field emission displays (FED) [2,3]. Especially as cathodoluminescent materials, as adsorbent materials [4,5,6], as sensitive materials for sensor devices [7,8], or as photocatalysts for dye degradation [9]. The properties of these materials depend on their morphology, size, specific surface area, composition, and conductivity. An increased attention is also given to the selection of the host matrix and to the guest active center (luminescent, emitting, or recognizing). The appropriate guests can be mixed oxides, tetrapyrrolic macrocycles (especially porphyrins), or corroles. Much emphasis was placed on the optimization of the doping ratio of oxides in order to improve energy transfer, thermal properties [10], and color stability [11,12,13,14].

The pseudo-binary oxide Zn_3_Nb_2_O_8_ has been reported as a material with excellent luminous performance and even self-excited luminescence [15,16,17]. Zn_3_Ta_2_O_8_ oxides with layered crystal structures were already prepared by a nonconventional hydrothermal method, and their electronic-band structures, optical properties, and photocatalytic activities were investigated. For the obtained materials, the UV–Vis diffuse reflectance spectra revealed that Zn_3_Ta_2_O_8_ exhibited band gaps of 4.5 eV [18]. From the electronic band structure calculations using the DFT method, it was found that the valence band was constructed by the hybridization of Zn 3d and O 2p orbitals, whereas the conduction band consisted of Ta 5d (Zn_3_Ta_2_O_8_) orbitals [19,20].

Quanto-chemical descriptors [21] were able to analyze the involved mechanisms, no matter if molecular, macromolecular, or solid-state structures are considered. As a consequence, the prediction and properties’ design of the pseudo-binary oxides became an intrinsic stage of the development for different applications, such as solar cells [22,23,24], piezoelectronics for sensors [25], hydrogen-storage materials [26], solid-state batteries [27], and controlled explosives [28].

Previous research showed that hybrid materials between mixed oxides such as Zn_3_Nb_2_O_8_ and a large plethora of porphyrins are useful in the corrosion inhibition of steel in different media (acid or salted) based on the involved synergy [29,30,31].

On the other hand, the porphyrin-silica materials exhibit high synergy as adsorbent materials for CO_2_ gas [32,33] or for different dyes, such as fuchsine B [34], methylene blue [33], and Congo red [35].

**Scheme 1:**
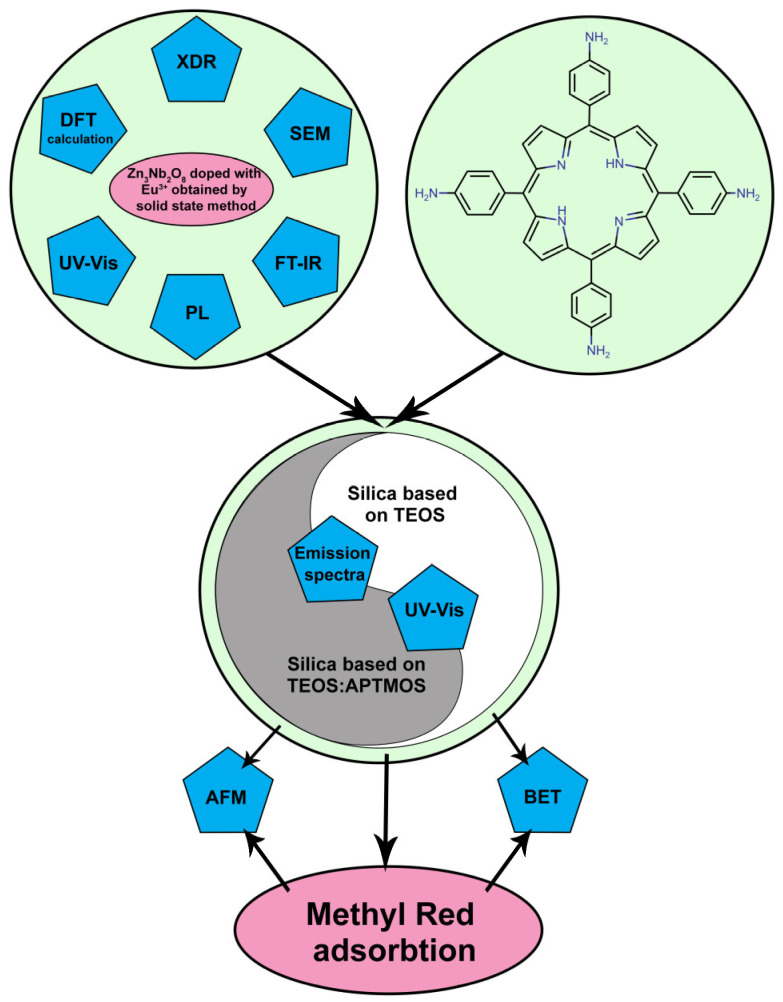
The workflow of the main research steps.

In the present work, we were concerned with obtaining a three-partnership nanomaterial composed of silica matrices, Zn_3_Nb_2_O_8_ oxide doped with Eu^3+^, and an amino-substituted porphyrin to highlight the synergistic effect towards methyl red removal and discoloration from wastewaters. We present a workflow containing the main steps performed in this research in Scheme 1.

**Figure 1:**
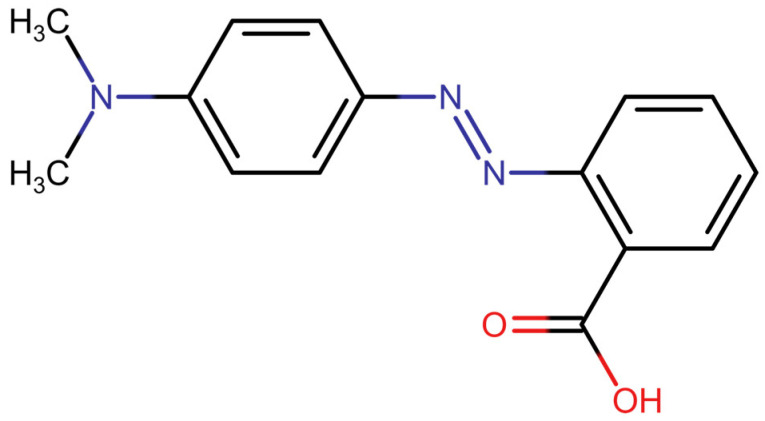
Chemical structure of methyl red.

We target methyl red (MR, Figure 1) removal from wastewaters because it is a pollutant that can cause major neurochemical damage to humans, allergies, irritations, infections of the eyes or skin, and infections of the digestive tract [36]. More than these, its oxygen-biodegraded products, 2-aminobenzoic acid and N-N-dimethyl-p-phenylene diamine, are mutagenic [37]. MR is an acidic azo dye used as a pH indicator [38], in the textile, paper, and paint industries, and also as a dopant for improving the electrical properties of nematic liquid crystal cells [39].

Since then, the degradation of MR in 98.20% percentage has been performed using Fe_3_O_4_-Fe_2_O_2_@SiO_2_ obtained from natural sources and H_2_O_2_ as oxidizers [40]. A quantity of 100 mg catalyst produces, in the presence of hydrogen peroxide, hydroxyl radicals capable of degrading MR at a pH = 3 in 180 min of exposure.

Another method to decolorize MR-contaminated waters is the use of *Bacillus thuringiensis* RI16 in static conditions [41] or *Pseudomonas aeruginosa* [42]. This strain proved an 81.49% degradation efficiency in optimized conditions at pH 9 and 3 days of incubation.

The classical method for dye removal is adsorption. Table 1 presents some of the most recent adsorbent materials for the elimination of methyl red from wastewater. As can be seen in Table 1, the adsorption capacity varies largely from 2.15 mg/g to 672.7 mg/g [43,44,45,46,47,48,49].

## 2. Results

### 2.1. Characterization of Zn_3_Nb_2_O_8_: Non-Doped and Doped with Eu^3+^ Ions

**Figure 2:**
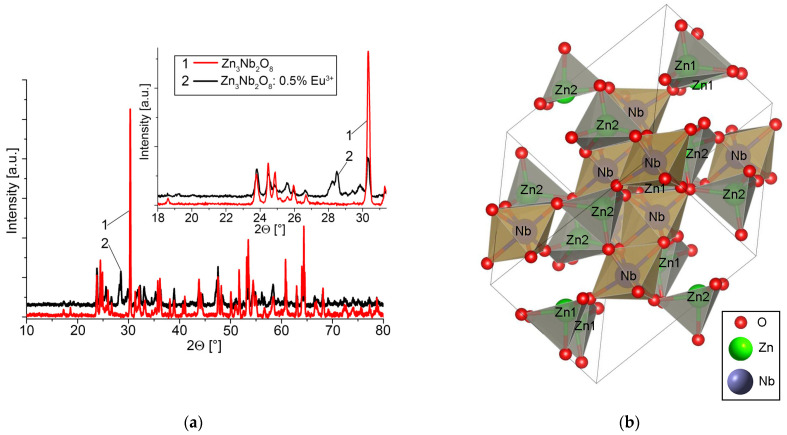
XRD patterns of: (**a**) 1—Zn_3_Nb_2_O_8_ and 2—Zn_3_Nb_2_O_8_: 0.5% Eu^3+^. A magnified zone of the X-ray diffraction spectrum containing the most important peaks is inserted. (**b**) Primitive cell of Zn_3_Nb_2_O_8_ determined with the CRYSTAL14 software.

Figure 2a presents the XRD patterns of the pseudo-binary oxide Zn_3_Nb_2_O_8_ nanomaterials, non-doped and doped with Eu^3+^, and a monoclinic phase of the Zn_3_Nb_2_O_8_ belonging to the C2/c space group (number 15) is revealed. Both nanomaterials were identified using JCPDS, card no. 01-079-1164, in the (−511) plane that is attributed to the highest intensity peak 2θ = 30.32°. Based on X-ray diffraction analysis using the Full Prof Suite computer package, the data lattice constants (Miller indices) for Zn_3_Nb_2_O_8_ were calculated: a = 9.99 Å, b = 9.99 Å, c = 5.22 Å, α = β = 90°, γ = 145.5°, and the elementary cell volume V = 583.48 Å^3^. In monoclinic symmetry (the C2/c space group), Zn_3_Nb_2_O_8_ forms a complex polyhedral structure with tetrahedral [ZnO_4_] and octahedral [NbO_6_] geometry (Figure 2b) [50]. Each NbO_6_ octahedron (Figure 2b) layer is located in-between Zn(1)O_4_ and Zn(2)O_4_ tetrahedron layers by edge sharing, which forms an “O” type arrangement. The Zn(1) and Zn(2) cations prefer to occupy two 4 e symmetric irreducible sites of C_2_ symmetry, while the Nb and O ions prefer to occupy the 8 f Wyckoff’s positions [51], having *C_1_* symmetry [50].

When the pseudo-binary oxide Zn_3_Nb_2_O_8_ nanomaterials were doped with Eu^3+^ ions, a shift to the smaller 2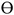 values for the entire XRD spectrum was observed. The shifting of the peak is mainly due to the difference in ionic radii between the initial element and the Eu^3+^ dopant ion, which introduces different parameters in the lattice [52,53].

Using density functional theory (DFT) with the CRYSTAL14 computer code [54,55,56,57], the preferred occupancy of Zn or Nb in the crystalline sites was calculated. Only the valence electrons were taken into account, the others playing the role of a screen for the charge of the nucleus, resulting in the use of the effective core pseudopotential (ECP). All the crystallographic sites have to be assumed to be fully occupied.

Using the same computer code [58], the ionic configuration of the O^2−^, Nb^5+^, and Zn^2+^ ions was established. The primitive cell comprises (Figure 2b) in its asymmetrical unit positions, irreducible in terms of structure symmetry, seven types of ions, as follows: Nb, Zn1, Zn2, and O1–O4. Consequently, Zn occupies two distinct positions from a symmetrical point of view, and O occupies four such positions that might determine the character of physical-chemical properties. In total, the primitive cell of the Zn_3_Nb_2_O_8_ crystalline structure contains four Nb^5+^, six Zn^2+^, and sixteen O^2−^ ions.

#### 2.1.1. Mulliken Analysis of Electron Populations

The Mulliken electron population analysis [59,60] reveals, first of all, a significantly different distribution of the charge of the 300 electrons in the primitive cell among its 26 ionic constituents. If the Nb ion is assigned a partial charge of 10.83 electrons, around the Zn ion there is a much denser electronic spatial charge of 18.8 electrons. Oxygen, with nine electrons distributed within the crystalline structure, is much closer to its oxidation state in the molecular binary combinations. They will find their place on ionic oxygen levels as valence electrons, as can be seen in Table 2.

Mulliken analysis of the electron population was also performed by overlapping the orbitals of two neighboring atoms. The low values highlighted the high degree of ionic character of the chemical bonds in the crystalline lattice as well as the relative strength of the bonds between different atoms in the elementary cell, proving that the Nb-O bond is stronger than the Zn-O one. Zn-Zn overlays of [–0.001] [61,62,63] and those in the range [−0.05–0.025] for O_j_-O_j_ are all negative values, revealing the very low possibility of ionic rejection (Table 3).

#### 2.1.2. Scanning Electron Microscopy Analysis of Pseudo-Binary Oxides

**Figure 3:**
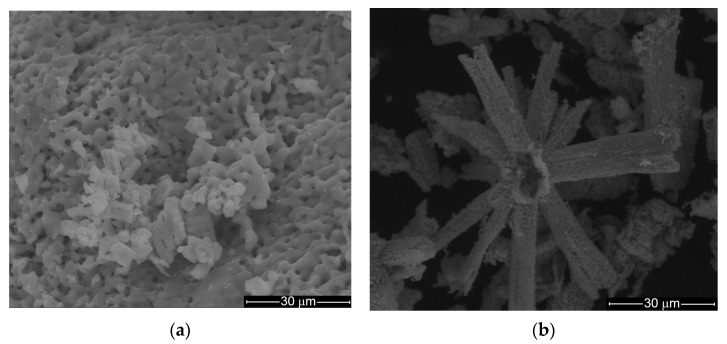
SEM images for (**a**) Zn_3_Nb_2_O_8_ and (**b**) Zn_3_Nb_2_O_8_: Eu^3+^.

Figure 3 shows the SEM morphology of the non-doped Zn_3_Nb_2_O_8_ and Eu^3+^-doped Zn_3_Nb_2_O_8_: Eu^3+^ oxides obtained by the solid-state method. The solid-state method was chosen due to its advantages, such as homogeneity and purity of the nanomaterials and a low reaction time. The magnification used in the SEM analysis was 1600× in a low vacuum. As can be seen from Figure 3a, when the pseudo-binary oxide Zn_3_Nb_2_O_8_ is not doped, it forms sponge-like agglomerates, while when it is doped with Eu^3+^ ions, it crystallizes in the form of long, thin platelets (Figure 3b) that organize into radial multiple-spoke wheels.

#### 2.1.3. Infrared Spectroscopic Characteristics of the Zn_3_Nb_2_O_8_ Nanomaterials

**Figure 4:**
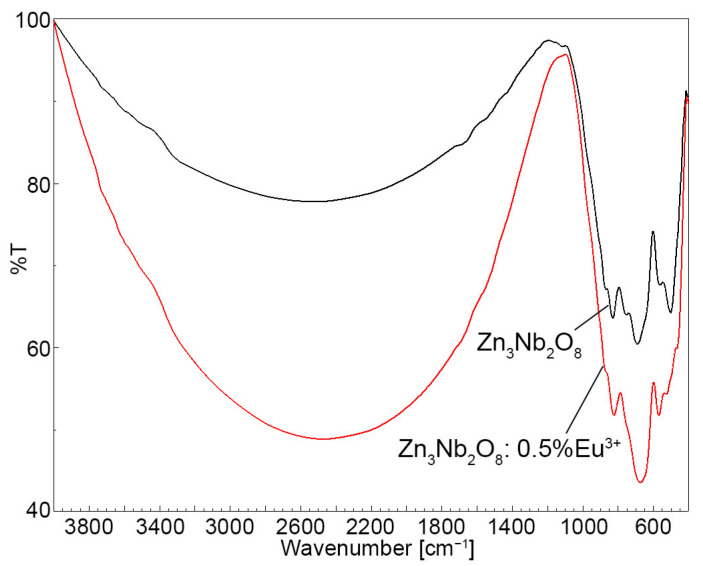
FT-IR spectra of the obtained pseudo-binary oxides: Zn_3_Nb_2_O_8_ and Zn_3_Nb_2_O_8_: 0.5% Eu^3+^ in KBr pellets.

From the FT-IR spectra of pure Zn_3_Nb_2_O_8_ and Eu^3+^ doped oxide (Figure 4), it can be observed that a shoulder is formed at 449 cm^−1^ and another one at 735 cm^−1^, not typical for either Nb_2_O_5_ or ZnO or Eu_2_O_3_ [64].

The absorption peak at 619 cm^−1^ corresponds to Zn–O bond stretching vibrations [65,66]. A band located at 501 cm^−1^ is assigned to ν(Zn–O–Nb) vibration [67], and the one at 563 cm^−1^ is due to ν_3_(Nb–O) vibration [68]. The peaks located at 691 cm^−1^ and at 829 cm^−1^ can be assigned to symmetric stretching of ν(Nb–O–Nb) [68,69] and to asymmetric stretching of ν(Nb=O) bonds [69]. The signal between 829−990 cm^−1^ is caused by stretching of the Zn-O bond [70].

The FT-IR spectrum for Zn_3_Nb_2_O_8_: Eu^3+^ presents a higher wavenumber for the stretching band belonging to Nb-O-Nb, meaning that the bond strength is increased due to the doping with Eu^3+^ ions.

The advantage of the Eu^3+^ ion insertion is that, because it has an even number of 4 f electrons, the beginning levels of the transitions in both the luminescence and the absorption spectra are nondegenerate (J = 0), and the interpretation of the ending transition levels is facilitated by the small total angular momentum *J* of the spectrum. The number of lines noticed for the ^5^D_J_→^7^F_0_ transitions in the absorption spectra or the ^5^D_0_→^7^F_J_ (J = 0–6) transitions in the luminescence spectra allows us to determine the site symmetry of the Eu^3+^ ions. The very intense and highly sensitive transition ^5^D_0_→^7^F_2_ indicates that the Eu^3+^ is not at the same site with a center of symmetry [71].

#### 2.1.4. Luminescence Spectra of Pseudo-Binary Oxides: Zn_3_Nb_2_O_8_ and Zn_3_Nb_2_O_8_: 0.5% Eu^3+^

**Figure 5:**
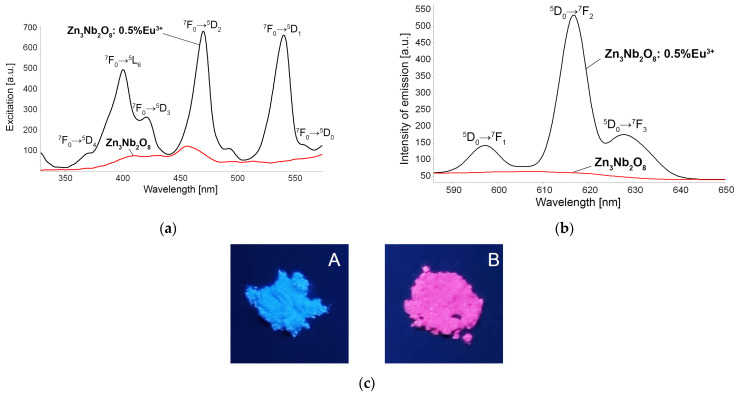
Photoluminescence (PL) spectra at room temperature (**a**) excitation; (**b**) emission of the synthesized nanomaterials of Zn_3_Nb_2_O_8_ and Zn_3_Nb_2_O_8_: 0.5% Eu^3+^; and (**c**) photos taken under UV irradiation at the wavelength λ = 254 nm: A—Zn_3_Nb_2_O_8_ and B—Zn_3_Nb_2_O_8_: 0.5% Eu^3+^.

The photoluminescence spectrum (PL) (Figure 5) consists of two emission bands due to the transitions: ^5^D_0_→^7^F_J_ (J = 1–3) and ^5^D_0_ →^7^F_J_ (J = 4), as well as the ^5^D_0_→^7^F_0_ (forbidden transition), due to the impurity ion Eu^3+^. From the experimental PL spectra of the Zn_3_Nb_2_O_8_: 0.5% Eu^3+^ nanocrystals (Figure 5a,b), the positional symmetry of the Eu^3+^ ions in the host matrix can be established using the selection rules from group theory [72]. It is known that, due to the absence of the center of symmetry in the host matrix (caused by the 4 f orbital with the opposite even orbital), the appearance of electric dipole transitions premises ^5^D_0_→^7^F_J_ (J = even) might result, while the presence of the center of symmetry for impurity ions Eu^3+^ in the host matrix allows magnetic dipole transitions ^5^D_0_→^7^F_J_ (J = odd). The asymmetry ratio R [73], defined as the relative intensity of the electric dipole transition ^5^D_0_→^7^F_J_ (J = even) and the magnetic dipole transition ^5^D_0_→^7^F_J_ (J = odd), depends on the local symmetry of Eu^3+^ ions. When Eu^3+^ ions occupy the central inversion sites, the transition ^5^D_0_→^7^F_J_ (J = even) should be relatively strong, while ^5^D_0_→^7^F_J_ (J = odd) is partially forbidden and should be relatively weak. Thus, experimentally, the intensity ratio R = ^5^D_0_→^7^F_2_/^5^D_0_→^7^F_1_ is a measure of the degree of distortion from the local inversion symmetry of the Eu^3+^ ion in the network. For the calculation of the intensity ratio, we used the peak areas of the transitions ^5^D_0_→^7^F_1_ and ^5^D_0_→^7^F_2_, respectively, for Eu^3+^. The intensity ratio R for Eu^3+^ is 2.6; thus, the local symmetry of Eu^3+^ ions in the Zn_3_Nb_2_O_8_ host matrix prefers to occupy the tetrahedral (Zn^2+^) or octahedral (Nb^5+^) symmetry without an inversion center. The forbidden transitions ^5^D_0_ → ^7^F_0_ are due to the crystal field effect [74], indicating that some impurity ions of Eu^3+^ are found in an interstitial place with low octahedral symmetry.

The emission spectra of Zn_3_Nb_2_O_8_ proved that this is an intrinsic blue light emitter, but in the case of Zn_3_Nb_2_O_8_: 0.5% Eu^3+^, an intense emission in the red region is produced due to the ^5^D_0_→^7^F_1,2_ that are hypersensitive transitions, being highly influenced by the coordination environment of Eu^3+^ ions [75] (Figure 5c).

### 2.2. UV-Vis Characterization of TAPP Porphyrin, Zn_3_Nb_2_O_8_, and Zn_3_Nb_2_O_8_: 0.5% Eu^3+^

**Figure 6:**
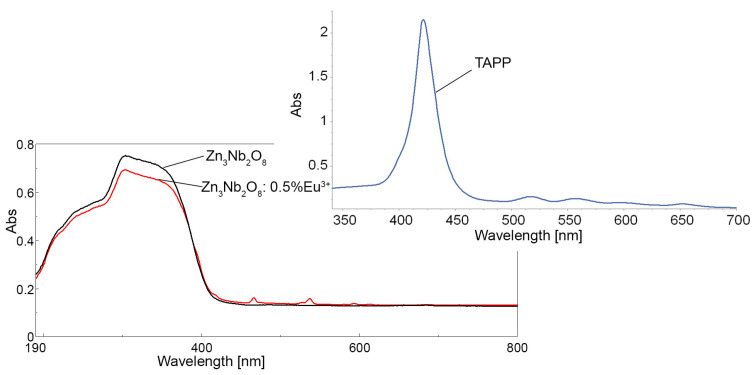
Overlapped UV-Vis absorption spectra for solid registration of Zn_3_Nb_2_O_8,_ Zn_3_Nb_2_O_8_: 0.5% Eu^3+^, and for tetrakis-(4-amino-phenyl)-porphyrin (TAPP) in DMSO.

The most significant band of porphyrin is the Soret band, or B band, located around 422 nm and assigned to the electronic transition from A_1u_ orbitals to E_g_ empty orbitals. In the visible region from 500 to 650 nm, the TAPP porphyrin presents four Q absorption bands (Figure 6). The differences in intensity of these bands depend on the nature of substituents grafted on the pyrrolic ring and are of etiotype in this case, meaning that the intensity is decreasing in the following order: OIV > QIII > QII > QI. The Q bands are assigned to electronic transitions of inner nitrogen atoms of porphyrin from the fully occupied electronic orbitals of A_2u_ to the empty orbitals belonging to the E_g_ electronic configuration [76].

**Figure 7:**
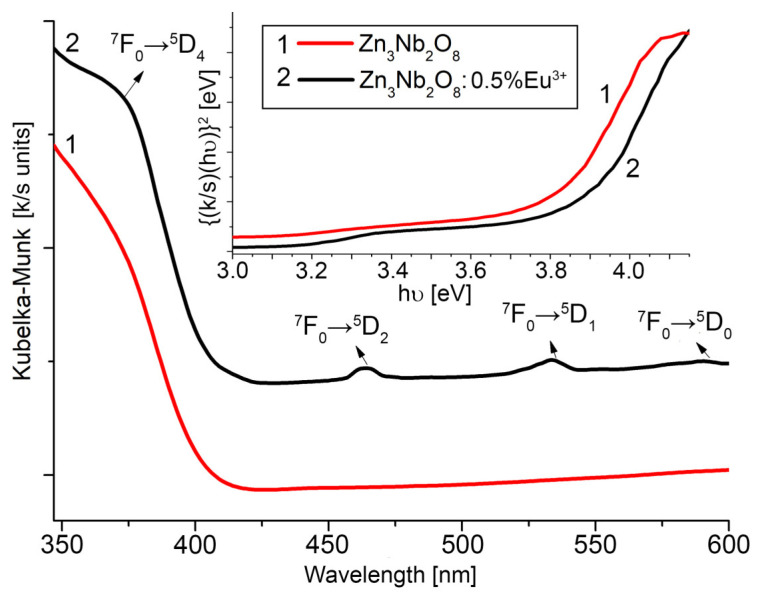
Absorption spectra of 1—Zn_3_Nb_2_O_8_ and 2—Zn_3_Nb_2_O_8_: 0.5% Eu^3+^. Inset: plot of {(*k*/*s*)*h*ν}^2^ vs. *h*ν (energy) of 1—Zn_3_Nb_2_O_8_ and 2—Zn_3_Nb_2_O_8_: 0.5% Eu^3+^.

For absorption spectra investigations, the integrating sphere of the UV-VIS-NIR Lambda 950 spectrophotometer was used, and the diffuse reflectance spectra were obtained at room temperature. From the absorption spectrum presented in Figure 7, it is observed that all the obtained nanocrystals have an absorption band in the UV range. The non-doped Zn_3_Nb_2_O_8_ has an absorption band at a wavelength of 304 nm, and this absorption band is slightly hypsocromically shifted when the nanocrystal is doped with Eu^3+^ ions (302 nm).

The band gap is estimated from the graph ${\left\{\left(k/s\right)h\nu \right\}}^{2}$ versus *h*ν (energy) in Figure 7, where k is the absorption coefficient, s is the diffusion coefficient, and *h*ν is the photon energy. The band gap for Zn_3_Nb_2_O_8_ is 3.7 eV, and for Zn_3_Nb_2_O_8_: 0.5% Eu^3+^, it is 3.85 eV. The absorption band is due to the f-f electronic transitions of Eu^3+^ ions from the ^7^F_0_ fundamental level to different excited states (^5^D_4_, ^5^D_2_, ^5^D_1_, and ^5^D_0_). If multiple individual transitions between energy levels are occurring, UV-Vis spectral lines are present, and if a sum of simultaneous transitions between very close energy levels is taking place, spectral bands can be discussed, as in this case.

The optical transitions that occur in the case of Eu^3+^ ions originate from the same configuration (4 f for trivalent Eu^3+^ ions) [77]. Quantum mechanics shows that various kinds of isolated microsystems can make transitions between discrete levels of energy. As it is clearly known, transitions to higher vibrational and rotational energy levels take place with energy absorption, and those to lower levels are accompanied by radiation emission. A triplet state (electrons have parallel spins) is lower in energy than the corresponding singlet state (electrons have paired spins). Furthermore, spin-forbidden and symmetry-forbidden selection rules have to be considered [78].

#### 2.2.1. Theoretical DFT Calculation for Zn_3_Nb_2_O_8_

**Figure 8:**
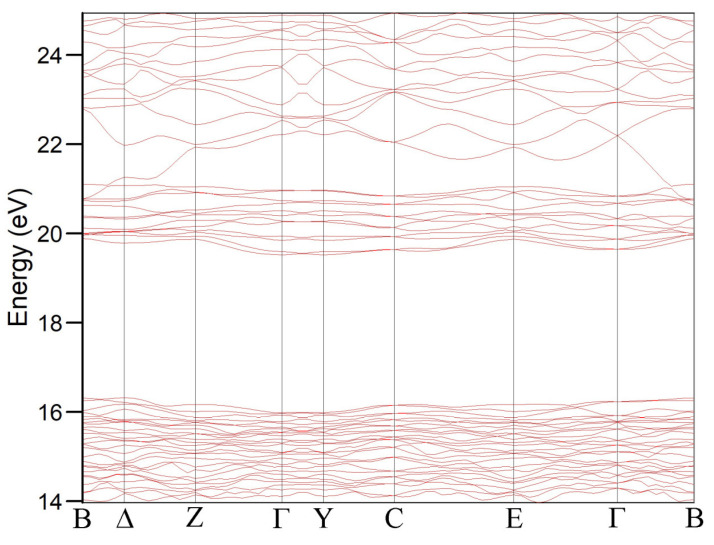
The gap between valence and conduction bands in Zn_3_Nb_2_O_8_.

For the calculation of the band structure of Zn_3_Nb_2_O_8_, we used the DFT method. In the primitive cell, there are 300 electrons distributed in 150 of the crystal orbitals. The limits of the band gap are between orbital 150 of the valence band, as the last occupied one, and orbital 151 of the conduction band, as the first unoccupied one. The calculated value of the band gap is about 3.7 eV, in accordance with the experimentally determined value (Figure 8).

**Figure 9:**
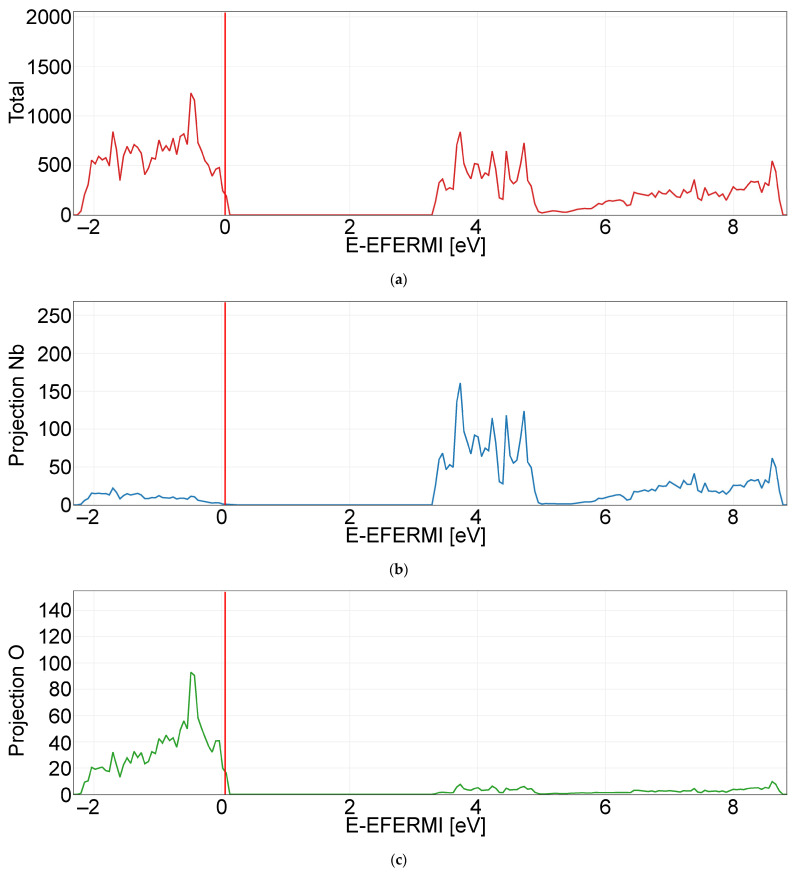

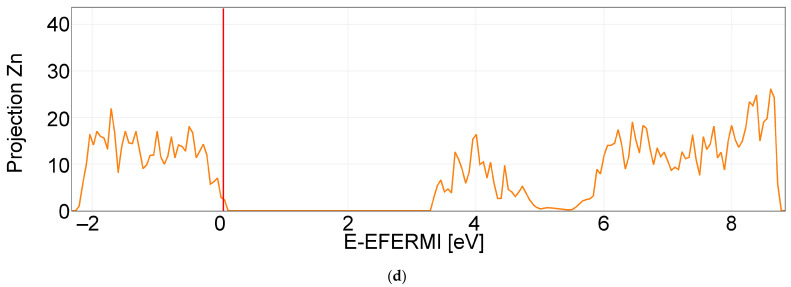
The projection of the density of electronic states (PDOS) as a function of the type of ions in the crystalline lattice. (**a**) Total density of electronic states in the valence band and conduction band of Zn_3_Nb_2_O_8_; (**b**) density of electronic states for the Nb ion; (**c**) density of electronic states on the O ion; (**d**) density of electronic states on the Zn ion.

Figure 9 presents the total densities of the electronic state function (Figure 9a) and the same for each of the three types of ions constituting the lattice. In Figure 9b, it is observed that Nb has a minor contribution in the valence band compared to that in the conduction band due to electrons identified by previous Mulliken analysis assigned within the lattice. Similarly, in Figure 9c, the contribution of the oxygen ion, especially in the valence band, is revealed. Figure 9d shows that around the Zn ion there is an appreciable density of electronic states, given by those 18 electrons, as previously highlighted by the Mulliken analysis. All the values and conclusions that emerge are in good agreement with the previous DFT analyses [79].

#### 2.2.2. DFT Analysis of the Crystalline Structure Zn_3_Nb_2_O_8_ in Order to Dope with Eu^3+^: Interstitial vs. Substitutional Chemical Choice

The main criterion for determining the existence of a crystalline structure using the DFT is the determination of its stability state by the convergence of its energy towards a minimum value.

From the very beginning, it should be emphasized that calculating and designing a crystalline material using DFT methods is more difficult as the doping level is lower. In principle, the method is simple in the case of substitutional doping and more difficult in the case of interstitial doping, in which the position in the asymmetric unit of the cell must be assigned so that the symmetry operations might correctly reconstitute the structure as a whole. The difficult issue lies in the fact that at low concentrations of doping, a multiplication of the primitive cell is mandatory to lead to the construction of a supercell, preserving the initial symmetry in which the doping ion is introduced. In this case, the 26 atoms present in the primitive cell with the 300 afferent electrons will supply in the smallest symmetrical supercell 208 atoms with a prohibitive number of electrons, requiring an extremely high computing power. Thus, the method is only feasible in the case of structures and symmetries that involve a limited number of ions in the primitive cell. The specific logistical effort and the allotted time are unjustifiable in this case.

DFT analysis is required and allows estimating the place that the doping ion can occupy in the crystalline structure, constituting a complementary method to the experimental data of spectral type.

**Figure 10:**
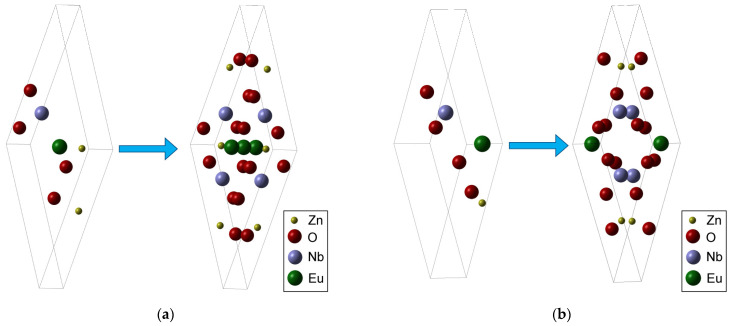
(**a**) Eu^3+^ in the interstitial position—the asymmetrical unit with the primitive cell; (**b**) Eu^3+^ in the substitutional Zn(1) position—the asymmetrical unit with the primitive cell.

Figure 10a,b show two types of positions of the Eu in the crystal lattice, equivalent in terms of the type of crystal lattice, both belonging to the same group of symmetry. They are practically achievable positions because, during the synthesis process, the two elements, Eu and Zn, react simultaneously and competitively with oxygen due to the fact that Eu gives up its electrons more easily than Zn. This is the reason why Eu will take its place in the lattice with a certain priority over Zn so that, from the morphostructural point of view, both its interstitial and substitutional positions will be, more or less, equally accessible to it.

Due to the fact that Eu^3+^ is coordinated in an octahedral system in EuO_6_, as it is in Eu_2_O_3_ used as a reactant, we are also suggesting the possibility of Eu^3+^ substituting Nb^5+^ ions in the lattice.

The DFT calculation of the Zn_3_Eu_2_O_8_ compound meets both the symmetry conditions of the lattice, in high similitude to that of Zn_3_Nb_2_O_8_, as well as the demanded requirement of convergence towards a minimum energy (major stability criterion), with a band gap of only about 2.5 eV, much smaller than the 3.7 eV that is the value of Zn_3_Nb_2_O_8_.

**Figure 11:**
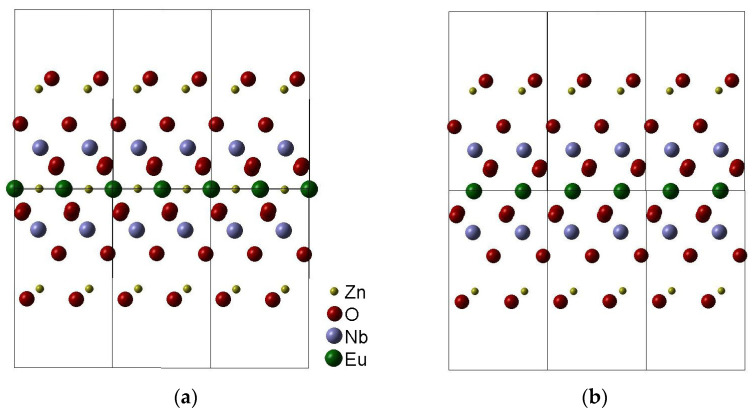
Eu^3+^ in the (**a**) interstitial or (**b**) substitutional Zn(1) position.

As can be seen in Figure 11, where three adjacent (primitive cells) were exposed in a favorable plane to a relevant observation, it can be noted that the size of the Eu^3+^ with ionic radius R = 0.947 Å does not cause a major differentiation in the process of occupying either of the two types of positions (Table 4).

Along with the geometric arguments given by the DFT analysis and presented in Figure 10a and Figure 11a and Table 4, justifying the interstitial position of Eu^3+^, the FT-IR spectrum for doped Zn_3_Nb_2_O_8_ (Figure 4) revealed an increasing wavenumber for the stretching band belonging to the Nb–O–Nb bond as compared to the length of the same bond in the un-doped lattice.

Therefore, in the case of doping, both variants are basically indiscernible in the XRD spectrum (Figure 2a), because the reticular planes are identical in position in the lattice. From the point of view of calculating the DFT for the above-mentioned structures, at a much higher concentration than that specific to doping, the energy convergence in the substitutional case was achieved, while in the interstitial case it was not. However, this fact is unquestionably caused by the interactions between the positive Eu^3+^ ions placed in the median plane of the elementary cell, which are too close to each other.

Thus, in the case of syntheses at higher concentrations, according to the DFT analysis, only the structure shown in Figure 11b is possible. In this case, which is not doping but a structure of the type Zn_3-x_Eu_x_Nb_2_O_8_, the band gap is canceled, the material becoming a conductor. The Eu^3+^ ion contributes to the lattice with a large number of conduction electrons, but the lattice is, according to the calculation results, indeed possible.

In the case of low concentrations, as in this case, where these interactions on the middle area are practically non-existent, both types of positions in the lattice are available without canceling the band gap.

### 2.3. Characterization of Silica Materials by UV-Vis Spectroscopy

The resulting silica materials were ground to a final fineness ~10 μm and analyzed by UV-Vis and fluorescent spectroscopy in the solid state.

**Figure 12:**
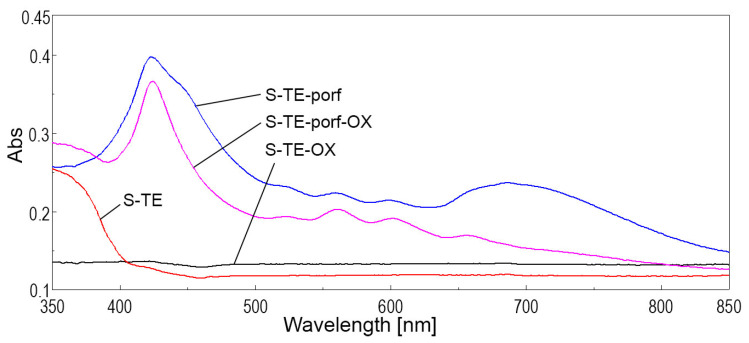
Overlapped UV-Vis spectra in the solid state for silica samples obtained from solely TEOS.

**Figure 13:**
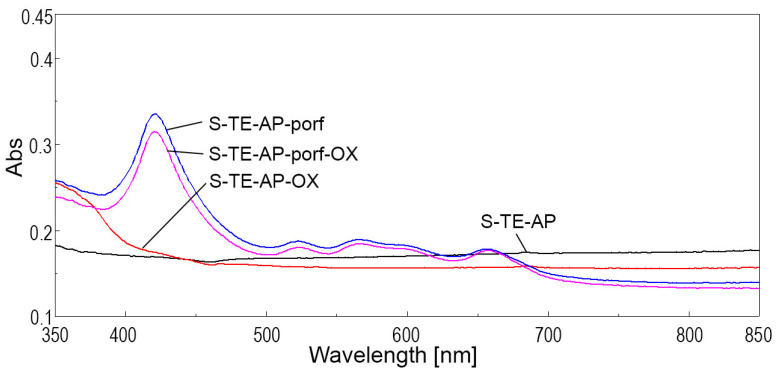
Overlapped UV-Vis spectra in the solid state for silica samples obtained from TEOS and APTMOS precursors in a 9:1 molar ratio.

By analyzing the overlapped spectra for solid silica samples containing porphyrin alone, pseudo-binary oxide alone, or a mixture of the two materials, shown in Figure 12 and Figure 13, it can be noticed, as expected, that both types of silica materials containing porphyrin (samples S-TE-porf and S-TE-AP-porf) present the highest absorption intensity at 423 nm.

The synergism between TAPP porphyrin and the TEOS-based and TEOS-AP based silica matrices is related, along with the hyperchromic effect, to the widening of the visible domain of absorption. In comparison, the presence of Zn_3_Nb_2_O_8_ doped with 0.5% Eu^3+^ in S-TE-porf-OX (Figure 12) and S-TE-AP-porf-OX (Figure 13), slightly diminishes both of these effects.

The UV-Vis spectra performed in solid state for the two types of silica materials containing TAPP porphyrin (Figure S1) were also analyzed and are presented in the Supplementary Material.

### 2.4. Characterization of Silica Materials by Fluorescence Spectroscopy

The emission spectra for solid samples were registered using a wavelength λ_ex_ = 400 nm, an excitation slit = 15 nm, and an emission slit = 5 nm.

A completely different behavior than in UV-Vis spectra can be observed in fluorescence spectra, where the major influence is given by Zn_3_Nb_2_O_8_ doped with Eu^3+^. The widest and most intense band belongs to the emission of the S-TE-OX sample.

The location and intensity of the silica samples, apart from the nature of the hybrid components, are dependent on the sample porosity. It is known that the porosity characteristics of the samples influence the number and location of the emitting centers and also the OH-groups on the silica surface [80]. Studies in the field established that the increasing pore size of a silica material determines the decrease of its hydrophilic character because the distance between different species of Si-OH groups is larger [81].

**Figure 14:**
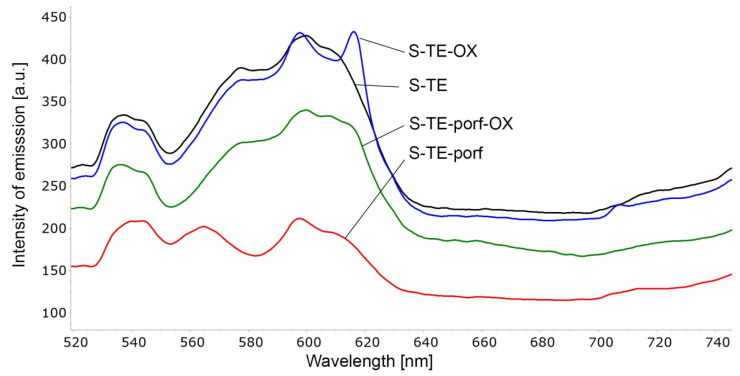
Overlapped emission spectra for solid silica samples based on TEOS: λ_ex_ = 400 nm, excitation slit = 15 nm, emission slit = 5 nm, 100 nm/min.

Except for the S-TE-OX sample containing Zn_3_Nb_2_O_8_ doped with Eu^3+^ that has the highest intensity of emission at 616 nm (Figure 14), the same as in the pseudo-binary oxide, all the other silica samples show a progression of peaks from 540 nm to 560 nm and 580 nm, with the main peaks positioned around 600 nm [82].

This situation clearly demonstrated that the emission of TEOS-based silica hybrid materials is dominated by TEOS-based silica emitting centers, no matter the incorporated dye or oxide.

**Figure 15:**
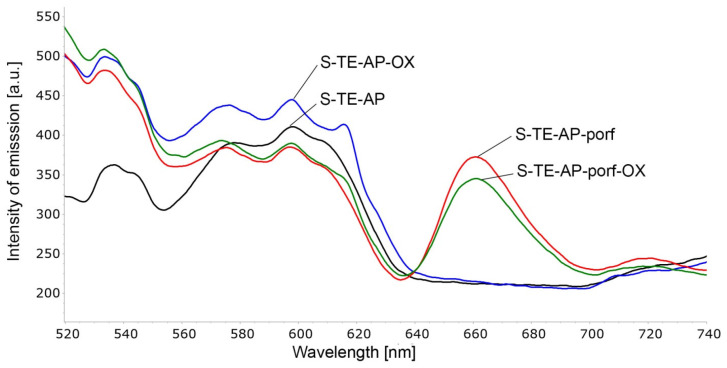
Overlapped emission spectra for solid silica samples containing the TEOS and APTMOS precursors in a 9:1 molar ratio: λ_ex_ = 400 nm, excitation slit = 15 nm, emission slit = 5 nm, 100 nm/min.

In Figure 15, the emission spectra of the materials based on TEOS and APTMOS precursors in a 9:1 molar ratio present the same progression of bands from 540 nm to 600 nm as in the case of TEOS-based silica. What differentiates these sets of spectra is the presence of intense emission bands due to the presence of TAPP porphyrin, as expected: a large and intense Q(0,0) band around 660 nm is associated with a lower intensity band in the red region around 720 nm assigned to Q(0,1) [32,83,84]. This widened type of emission from green to yellow, orange, and finally red extended the possibility of applications of these two materials: S-TE-AP-porf and S-TE-AP-porf-OX, which are highly present and reported in Eu^2+^ activated oxides [85], but the effect is also present and in an improved way in the sample that does not contain Eu^2+^ ions, namely: S-TE-AP-porf. This situation can be explained by the different polarity inside the silica pores when the AP precursor is the bridge between TAPP porphyrin and the pore walls. In such lower-polarity’ environments, the light emission process of the porphyrin is more facile [86].

So, it is clear that encapsulation of porphyrin dyes in silica matrices provides new photosensitive materials [87].

On excitation at 400 nm, the TAPP porphyrin shows two emission bands at 660 nm (Q(0,0) and another weaker emission at 720 nm (Q(0,1), corresponding to the S_1_→S_0_ transition. These bands are broadened and red shifted in the silica matrices as compared with the porphyrin spectrum in solution because of interactions with the silica network and differences in the environment. Furthermore, the aggregation seems to significantly contribute to the tendency toward red shifting of the bands [88].

As can be seen in Figure 15, the difference between excitation wavelength and emission wavelength for both S-TE-AP-porf and S-TE-AP-porf-OX samples, known as the Stokes shift, is larger than 240 nm, so that they can be considered isolated, which is a benefit for diminution of background signals for sensing applications [89].

**Figure 16:**
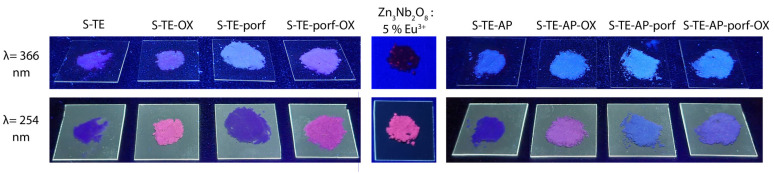
Color emission of the silica materials (under UV light) in comparison with the pseudo-binary oxide doped with Eu^3+^ ions.

Fluorescence (Figure 16) is generated by the fluorophore emission of a photon from the lowest excited state S_1_ to the ground state S_0_, in which simultaneously transitions from different vibrational levels will occur, having energies lower than those of the emitted photon [90].

### 2.5. Methyl Red (MR) Adsorption Investigations Using Hybrid Silica Materials

All the silica matrices obtained in this work were tested for their capacity to adsorb methyl red from synthetic water-based solutions. The concentrations of MR tested were 1 × 10^−4^ M and 1 × 10^−5^ M. The loadings of adsorbent material were 5 g/L, 10 g/L, and 20 g/L, respectively. The adsorption experiments were performed at room temperature (295 K) for an exposure time of 120 min.

The effect of pH was also tested by adjusting the MR solution c = 1 × 10^−4^ M to pH = 1.5 with HCl solution (c = 0.5 M) and to pH = 8.5 with NaOH solution (c = 0.5 M) with a loading of 2 g/L S-TE control adsorbent material. It was concluded that the natural pH of the MR solution (pH = 5.5) leads to the best adsorption.

The amount of adsorbed dye per unit of mass adsorbent, q_e_ [mg/g], was calculated according to Equation (1) [44]:

(1) $$q_{e}=\frac{c_{0}-c_{e}}{m}\times \;V$$

where $c_{0}$ = initial dye concentration [mg/L]

$c_{e}$ = final dye concentration at equilibrium [mg/L]

m = mass of adsorbent [g]

V = volume of dye solution [L]

The removal efficiency was calculated according to Equation (2):

(2) $$R.E.\;\left[\%\right]=\frac{c_{0}-{\;c}_{e}}{c_{0}}\times 100$$

These calculated results are presented in Table 5, Table 6 and Table 7.

**Figure 17:**
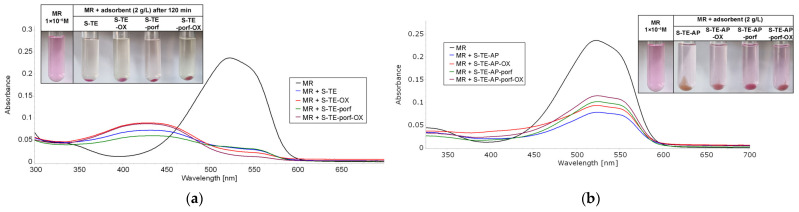
Overlaying UV-Vis spectra on the supernatant after 120 min of exposure to a 5 mL MR solution (c = 1 × 10^−5^ M) for a loading of adsorbent material of 2 g/L, based on (**a**) TEOS and (**b**) TEOS:APTMOS precursors.

**Figure 18:**
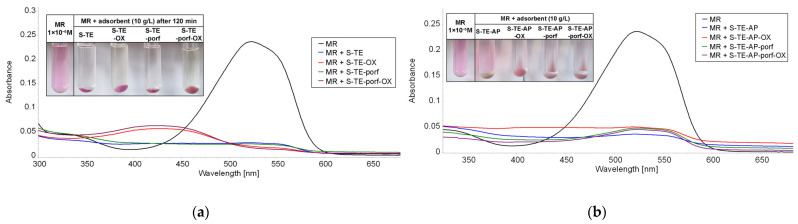
Superimposed UV-Vis spectra on the supernatant after 120 min of exposure to a 5 mL MR solution (c = 1 × 10^−5^ M) for a loading of adsorbent material of 10 g/L, based on (**a**) TEOS and (**b**) TEOS:APTMOS precursors.

**Figure 19:**
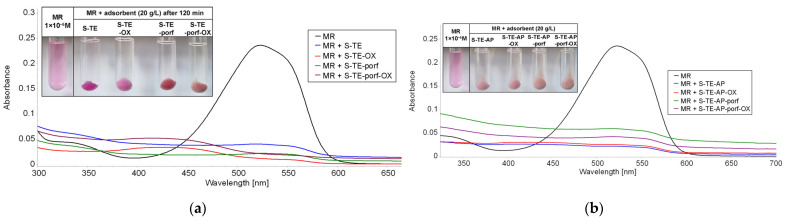
Superimposed UV-Vis spectra on the supernatant after 120 min of exposure to a 5 mL MR solution (c = 1 × 10^−5^ M) for a loading of adsorbent material of 20 g/L, based on (**a**) TEOS and (**b**) TEOS:APTMOS precursors.

The UV-Vis spectra given below (Figure 17, Figure 18 and Figure 19) illustrate the best results (adsorption and discoloration) obtained for water containing methyl red of concentration 1 × 10^−5^ M. Figure S2, Figure S3, Figure S4 from Supplementary Materials present the UV-Vis spectra of the supernatant after 120 min exposure to MR solution at higher concentration for different loadings of adsorbent materials.

A comparison between the performances of different adsorbents shows clearly that S-TE-porf-OX (at loadings of 2 g/L and 10 g/L—Figure 17 and Figure 18) is the best material capable of discoloring MR from water. When the loading was higher, namely 20 g/L, the best materials for MR discoloration were the hybrid silica materials based on TEOS silica matrices and the pseudo-binary oxide (Zn_3_Nb_2_O_8_). As a general rule, when TEOS-based precursors are used, the materials perform better.

**Table 6:** Total mass of adsorbed dye per unit mass of adsorbent and removal efficiency for a loading of 10 g/L.

	Methyl Red Concentration 1 × 10^−4^ M		Methyl Red Concentration 1 × 10^−5^M	
Adsorbent Material	q_e_ [mg\g]	R.E. [%]	q_e_ [mg\g]	R.E. [%]
S-TE (control)	1.625	60.36	0.238	88.54
**S-TE-OX**	**2.308**	**85.69**	**0.247**	**91.59**
S-TE-porf	1.620	60.17	0.241	89.58
**S-TE-porf-OX**	**2.463**	**91.46**	**0.250**	**92.87**
S-TE-AP (control)	1.005	37.33	0.228	84.79
S-TE-AP-OX	1.258	46.73	0.213	78.91
S-TE-AP-porf	1.031	38.30	0.215	79.79
S-TE-AP-porf-OX	0.918	34.09	0.218	80.93

**Table 7:** Quantity of adsorbed dye per unit mass of adsorbent and removal efficiency for a loading of 20 g/L.

	Methyl Red Concentration 1 × 10^−4^ M		Methyl Red Concentration 1 × 10^−5^ M	
Adsorbent Material	q_e_ [mg\g]	R.E. [%]	q_e_ [mg\g]	R.E. [%]
S-TE (control)	0.900	66.87	0.111	82.76
**S-TE-OX**	**1.169**	**86.78**	**0.128**	**94.82**
S-TE-porf	0.923	68.56	0.122	90.44
**S-TE-porf-OX**	**1.224**	**90.91**	**0.122**	**90.76**
S-TE-AP (control)	0.998	74.14	0.122	90.57
S-TE-AP-OX	1.006	74.68	0.120	89.02
S-TE-AP-porf	0.767	56.98	0.101	74.73
S-TE-AP-porf-OX	0.749	55.62	0.110	81.97

#### 2.5.1. Comparative Atomic Force Microscopy (AFM) Characterization of the Hybrid Silica Materials before and after MR Adsorption

**Figure 20:**
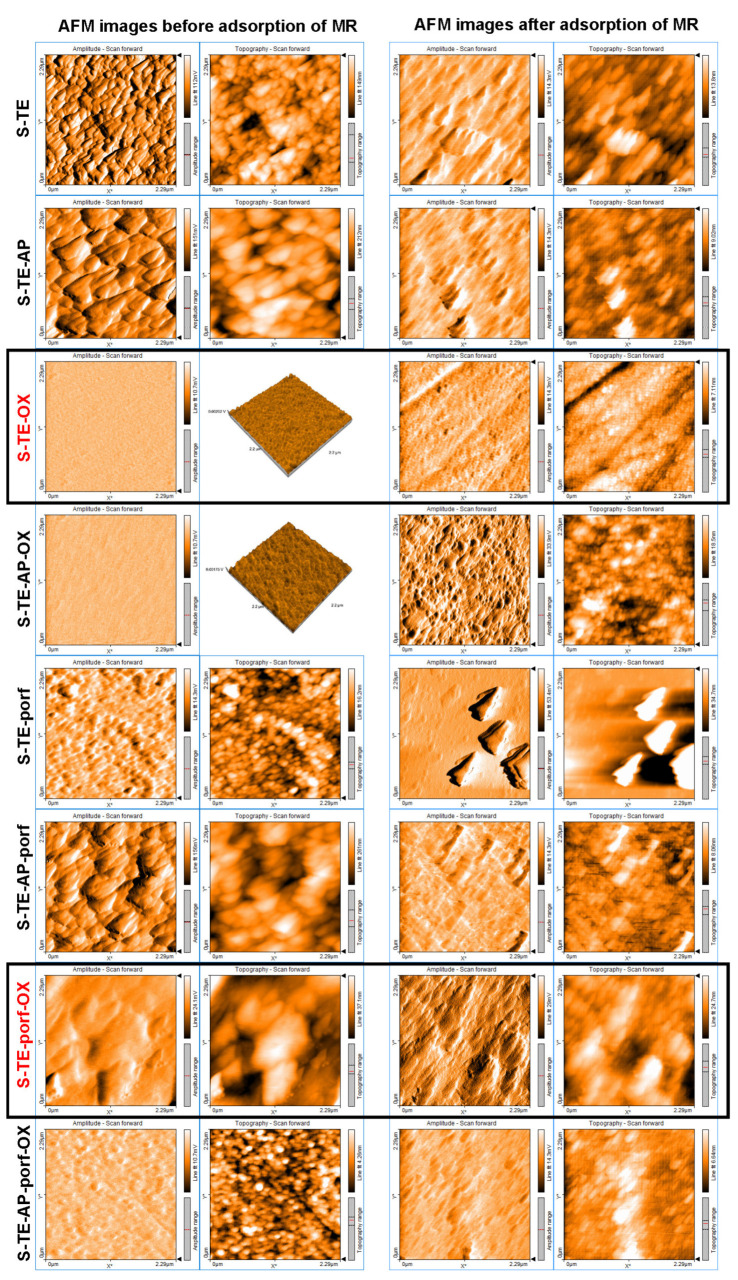
AFM characterization of the hybrid silica nanomaterials containing Zn_3_Nb_2_O_8_ doped with Eu^3+^ and amino-substituted porphyrin before and after methyl red (MR) absorption. The best adsorbent materials are highlighted in frames.

Figure 20 shows 2D AFM images (color map, 3D, and topography) recorded using a non-contact mode cantilever before and after methyl red (MR) absorption.

From the AFM investigations (Table 8), it can be seen that after absorption of MR, the hybrid silica particles underwent changes in their morphology as follows: the size of the aggregates decreased, displaying a novel rearrangement of the aggregates from triangular, well-defined shapes to elongated triangles or ovoids.

The smallest particle size is noticed in the case of the hybrid silica nanomaterials containing Zn_3_Nb_2_O_8_ doped with Eu^3+^ both before (12 and 28 nm) and after (3 and 7 nm) MR absorption.

Using the software Nanosurf Report v4 upgraded 2018 and calculations based on the equations reported in [91], the particle dimensions, the nanorugosity-S_a_ (average roughness), and S_q_ (square root roughness) were given for each sample (Table 8). The decrease in S_a_ (average roughness) and S_q_ (square root roughness) after MR adsorption is revealed, meaning that the porosity of all adsorbents is diminished and the materials are covered with a smoother, thinner layer of MR.

**Table 8:** The nanoroughness and particle dimensions of the hybrid silica nanomaterials containing Zn_3_Nb_2_O_8_ doped with Eu^3+^ and/or amino substituted porphyrin before and after methyl red (MR) absorption.

	Before Adsorption of MR			After Adsorption of MR		
Sample	Average Roughness (S_a_) [nm]	Square Root Roughness (S_q_) [nm]	Particle Dimensions [nm]	Average Roughness (S_a_) [nm]	Square Root Roughness (S_q_) [nm]	Particle Dimensions [nm]
S-TE	29.046	35.582	83	21.768	22.295	65
S-TE-AP	30.683	38.415	98	28.036	31.475	71
S-TE-OX	5.114	8.171	12	1.016	1.985	3
S-TE-AP-OX	9.125	12.149	28	2.697	3.485	7
S-TE-porf	25.214	29.906	75	14.992	15.337	53
S-TE-AP-porf	26.455	32.101	80	13.4691	14.489	58
S-TE-porf-OX	20.640	25.183	56	10.325	10.519	39
S-TE-AP-porf-OX	22.341	27.021	62	10.911	11.236	44

#### 2.5.2. BET Analysis

**Figure 21:**
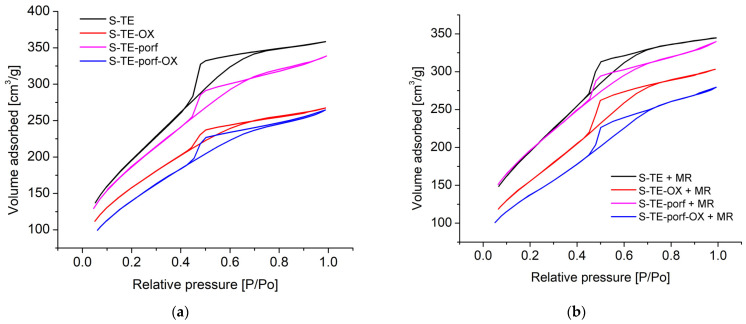
Nitrogen adsorption-desorption isotherms of samples based on TEOS (**a**) before and (**b**) after adsorption of MR.

Figure 21a,b show the nitrogen adsorption-desorption isotherms of samples based on TEOS before and after adsorption of MR.

Type IVa isotherms with H2a hysteresis, which are representative for samples with inkbottle-shaped pores, resulted both before and after adsorption of MR, based on nitrogen adsorption-desorption isotherms and comparison with IUPAC data [92]. Since the samples present narrow-pore necks, they accommodate the dye well [92].

The textural parameters for samples based on TEOS are presented in Table 9 before exposure to MB and in Table 10 after MB adsorption.

Each of the hybrid materials provides a highly specific surface area. The highest surface area was obtained for the control sample (S-TE), with a value of 726 m^2^/g and a total pore volume of 0.556 cm^3^/g. The smallest surface area and total pore volume among these studied materials were obtained for sample S-TE-porf-OX. Despite this fact, the specific surface area of S-TE-porf-OX is still high enough to achieve the best adsorption performance, a fact explained by the presence of porphyrin and its binding properties. In all samples, the pore size is around 3 nm, and the entry dimensions are similar, as was also observed in AFM measurements.

Correlating these data with the adsorption performances of MR on the samples based on TEOS, we observed that when the fractal dimension is increased, meaning that the rugosity is increased, the adsorption results are lower, as was also noticed from AFM data.

By comparison with the samples before adding MR, we observed that the tendency is to decrease the surface area. Thus, we can conclude that in this case, the MR is mainly adsorbed on the surface of the absorbent samples because the rugosity values decreased and the pore size distribution was almost the same.

**Figure 22:**
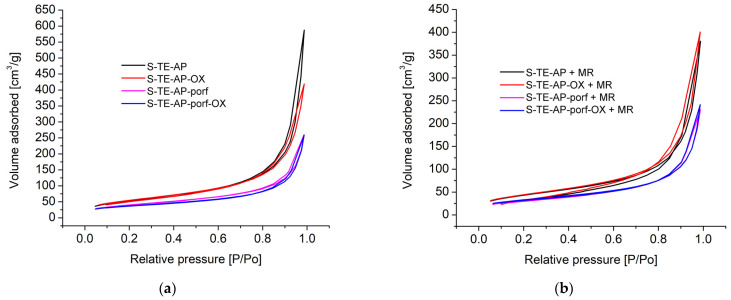
Nitrogen adsorption-desorption isotherms of samples based on TEOS:APTMOS (**a**) before and (**b**) after adsorption of MR.

Further, Figure 22a,b indicate the nitrogen adsorption-desorption isotherms for samples based on TEOS:APTMOS.

In the case of mixed silica precursors, nitrogen adsorption and desorption isotherms are type IVa with a H3 hysteresis. The hysteresis of specific type H3 is usually revealed by plate-like particles with a grooved-pore network consisting of macropores that are not completely filled with adsorbate [93]. The capillary condensation in these samples takes place near 0.8 P/Po, which confirms that the samples present macroporosity.

Table 11 shows the textural parameters of samples based on TEOS:APTMOS before exposure to MB and Table 12 after MB adsorption, respectively.

Analyzing the samples based on TEOS:APTMOS, we can conclude that the same situation as in the previous series occurs. The highest surface area was obtained for samples S-TE-AP and S-TE-AP-OX at around 198 m^2^/g. Even in this case, the samples with the smallest rugosity present the highest efficiency of adsorption. The main difference compared with the TEOS-based series of hybrid materials is that the surface area decreases almost four times and the pore size diameter increases considerably. Regarding the type of hysteresis, it might be concluded that in this case, the adsorption is due to MR entering the pores due to their open shape.

## 3. Materials and Methods

### 3.1. Materials and Reagents

The solid-state method [29,31,94] was selected due to its advantages, such as high purity, short reaction time, homogeneity, and low price, to obtain the Zn_3_Nb_2_O_8_ pseudo-binary oxide nanomaterials, both non-doped and doped with Eu^3+^ ions. Doping with Eu^3+^ ions improved the specific optical properties of the Zn_3_Nb_2_O_8-_based nanomaterials. The used precursors in the solid-state synthesis were: niobium pentoxide–Nb_2_O_5_ (99.9 %, Sigma-Aldrich, St. Louis, MO, USA); zinc oxide–ZnO (99.99 %, Merck KGaA, Darmstadt, Germany) in the molar ratio 3:1; and adding 0.5 % europium (III) oxide–Eu_2_O_3_ (99.9%, Sigma-Aldrich, St. Louis, MO, USA) as a doping ion to the Nb_2_O_5_ quantity. The synthesis was conducted in the calcination furnace SNOL (Telecomed SRL, Iasi, Romania) at a temperature of 1100 °C for 4 h at a rate of 5 °C/min for heating/cooling.

Tetraethyl orthosilicate (TEOS) and 3-aminopropyltrimethoxysilane (APTMOS) were provided by Fluka (Seelze, Germany); ethanol absolute (EtOH) was obtained from Chimreactivul SRL (Bucuresti, Romania); tetrahydrofuran (THF), hydrochloric acid (HCl), and ammonia (NH_3_) were purchased from Merck (Darmstadt, Germany); and all were purrum analyticum grade. The 5,10,15,20-tetra-aminophenylporphyrin (TAPP) was obtained and completely characterized in a previously published paper [95]. Methyl red originated from P OCh SA PPH Polskie Odczynniki Chemiczne (Gliwice, Poland).

### 3.2. Method for Obtaining Hybrid Silica Nanomaterials Containing Zn_3_Nb_2_O_8_ Doped with Eu^3+^ and/or Amino-Substituted Porphyrin

The Zn_3_Nb_2_O_8_ doped with 0.5% Eu^3+^ and/or the amino substituted porphyrin (TAPP) were immobilized in silica gels obtained by performing a two-step sol-gel process conducted in acid-base catalysis, starting from either solely tetraethoxysilane (TEOS) or two silica precursors: 3-aminopropyltrimethoxysilane (APTMOS) and TEOS, involved in 1/9 molar ratio.

In order to obtain the materials incorporating the oxide, the first step was to obtain the sol, which was conducted by acid catalysis using hydrochloric acid (HCl). The molar ratios between the silica precursor/precursors’ mixture/alcohol/water and HCl were chosen as 1:2:6:0.02, with the purpose of achieving low porosity and, as a consequence, high specific surface areas [32,96].

The second step, conducted in base catalysis, involves the previous sols by adding, under vigorous stirring, the finely ground to final fineness of ~20 μm of Zn_3_Nb_2_O_8_ doped with 0.5% Eu^3+^ (1% weight of the silica precursors) and the required amount of NH_3_ catalyst till the gelation occurs. After gelation, the gels were left to age for 24 h and then dried for 12 h at 125 °C.

The sol-gel samples containing tetrakis-(4-amino-phenyl)-porphyrin (TAPP), were similarly obtained using a 1/10,000 molar ratio between the silica precursors and the porphyrin, no matter if only porphyrin or both porphyrin and the pseudo-binary oxide were added in mixtures. 

Due to the known fact that porphyrin-based silica nanoparticles can suffer leakage when being dispersed in various organic solvents, TAPP porphyrin leakage was verified in water, ethanol, and tetrahydrofuran (THF), but no such phenomenon occurred.

In this way, eight samples were obtained. The named samples, combinations, and synthesis data are introduced in Table 13. As can easily be seen, the precursor type and the ratio between the two mixed silica precursors affect the time of gelation. The two control samples were obtained without immobilization of pseudo-binary oxide or porphyrin and were denoted as S-TE and S-TE-AP.

### 3.3. Apparatus

The crystallization phases of the pseudo-binary oxides Zn_3_Nb_2_O_8_ and Zn_3_Nb_2_O_8_ doped with Eu^3+^ nanomaterials were investigated by X-ray diffraction (model PW 3040/60 X’Pert PRO Powder Diffractometer (Malvern, UK)) with incident monochromatic Cu Kα (λ  =  1.5418 Å) radiation.

The field emission-scanning electron microscopy (SEM) model INSPECT S (Hillsboro, OH, USA) was performed at low vacuum, magnification = 1600 ×, weight distance = 11 mm, and high voltage = 25.00 kV. The morphology of the materials was analyzed by atomic force microscopy (AFM) using the NanoSurf ^®^EasyScan 2 Advanced Research (Liestal, Switzerland), scanned in noncontact mode with a scan size of 2 µm × 2 µm, time/line = 1 s, and points/line = 1024, and using the soft NanoSurf ^®^EasyScan 2 Advanced Research, the particle size and the nanoroughness (S_a_-the values of average roughness and S_q_-the mean square root roughness) before and after the adsorption of methyl red (MR) for each sample.

The band gap for Zn_3_Nb_2_O_8_ and Zn_3_Nb_2_O_8_ doped with Eu^3+^ nanomaterials was calculated using the diffuse reflectance spectra recorded at room temperature on the UV-VIS-NIR spectrometer Lambda 950 (Markham, ON, Canada).

The UV-Vis spectra were performed on a V-650-JASCO spectrophotometer (Pfungstadt, Germany). The liquid samples were recorded in a 10 mm wide quartz cuvette.

FT-IR spectra were carried out on a JASCO 430 FT-IR spectrometer (Hachioji, Japan), as potassium bromide pellets.

The mixer mill used for grinding silica samples is manufactured by Retsch GmbH, model MM 200 (Haan, Germany).

BET analysis investigated on a QuantachromeNova 1200 apparatus at 77 K provided nitrogen isotherms, from which the total pore volume (Vp), average pore diameter (Dp), and specific surface area (SBET) were calculated. The surface roughness was calculated by the Frenkel-Halsey-Hill (FHH) equations [97]. Before analysis, the samples were degassed at 55 °C in a vacuum for 8 h. The BET (Brunauer-Emmett-Teller) method was used to calculate the specific surface area [98], and the BJH (Barrett, Joyner, and Halenda) method provided the results for pore size distribution [33,97].

## 4. Conclusions

Because azo dyes are water pollutants known to cause, aside from allergies, major neurochemical damage to humans, a three-partnership was set up between two types of silica matrices (based on TEOS and TEOS/APTMOS), incorporating Eu^3+^-doped Zn_3_Nb_2_O_8_ oxide and a symmetrical amino-substituted porphyrin, presuming a synergistic effect towards methyl red removal and discoloration from wastewater.

To achieve this goal, the monoclinic phase of Zn_3_Nb_2_O_8_ belonging to the C2/c space group was obtained, and the emission spectra proved that this is an intrinsic blue light emitter. Doping the pseudo-binary oxide Zn_3_Nb_2_O_8_ with 0.5% Eu^3+^ generated an intense emission in the red region due to the ^5^D_0_→^7^F_1,2_ hypersensitive transition. Using the DFT method, the ionic configuration of the O^2−^, Nb^5+^, and Zn^2+^, the band gap, and the prediction of the position of Eu^3+^ doped in the crystal lattice were established.

A comparison between the non-doped pseudo-binary oxide Zn_3_Nb_2_O_8_ and its doped crystal with Eu^3+^ ions was performed by X-ray powder diffraction, Mulliken electron population analysis, scanning electron microscopy, infrared, luminescence, UV-Vis, fluorescence spectroscopy, atomic force microscopy, and the Brunauer-Emmett-Teller (BET) method.

An overview of the BET data indicates that TEOS-based silica materials are offering optimized conditions as adsorbents with very high specific surface areas (between 518 and 726 m^2^/g). In comparison, silica materials also containing APTMOS have four times lower specific surface areas (between 131 and 199 m^2^/g), which might be the reason for their decreased adsorption performance. Although the specific surface area of S-TE-porf-OX is only 518 m^2^/g, it is still high enough to achieve the best adsorption performance due to the significant contribution of the amino-substituted porphyrin that furnishes supplementary amino binding sites for MR. A great technical advantage is that the natural pH of the MR solution (pH = 5.5) leads to its best adsorption, avoiding any additional reagents or fixing operations.

Two different types of MR adsorption mechanisms can be reported: one implying surface absorbance in the case of TEOS-based materials and a second one involving the entry of the dye into the pores due to their open groove shape network in the case of silica materials also containing APTMOS.

## Figures and Tables

**Table 1:** The removal performance of MR from contaminated waters using various adsorbent materials.

Adsorbent Material	Removal Percentage	Adsorption Capacity [mg/g]	Conditions	Advantages	Disadvantages	Ref.
Bark of the *Dodonaea viscosa* (Hopbush) plant	73	36.64	pH = 1 75 min contact time	Inexpensive	Selective distribution of this plant in regions of Africa, the Americas, southern Asia, and Australasia Highly acidic pH	[43]
Nano-zero-valent iron dispersed in chitosan and sodium alginate	68.0	3.38	pH = 2 60 min exposure	-	-	[44]
Beads of waste foundry dust, sodium alginate, and polyethylene imine	89.1	672.7	pH = 9 3 h exposure	Easy to recover; Magnetic material;	Local industry foundry	[45]
Activated carbon obtained from the carbonization and activation of empty raw palm oil fruit bunches	86.72	2.15	pH = 2 180 min contact time	Reducing waste from the palm oil industry	Palm-oil production is perturbing the rainforest; Energy-consuming method for obtaining the adsorbent	[46]
Jute fibers treated with sodium carbonate	-	32.11	pH = 7.08 120 min contact time	Inexpensive	Local Indian plants	[47]
Charred *Shorea robusta* Saw-Dust modified with concentrated sulfuric acid or NaOH and CS_2_		70; 130	pH = 4 3–4 h contact time	-	Tedious preparation of adsorbent material	[48]
Hen feather	92	6.02	pH = 4 90 min exposure time	Effective; Low cost	-	[49]
TEOS-based silica material functionalized with tetra-aminophenyl porphyrin and Zn_3_Nb_2_O_8_ doped with Eu^3+^	88.04	11.85	pH = 5.5 120 min exposure time	No pH adjustments are necessary	-	This work

**Table 2:** Net electronic load.

	Nb	Zn1	Zn2	O1	O2	O3	O4
Nr. electrons/ion	10.831	18.818	18.837	9.037	9.075	8.943	8.868

**Table 3:** Overlapping coefficients of atomic orbitals in the lattice.

	O1	O2	O3	O4
**Nb**	0.076	0.106	0.112	0.065
**Zn1**	0.045	0.010	0.032	0.048
**Zn2**	0.013	0.038	0.066	0.021

**Table 4:** The distance between Eu^3+^ and the closest neighbors for the interstitial and substitutional positions.

	R [Å]				R [Å]		
Eu^3+^ interstitial	1.5302	O21	O23	Eu^3+^ substitutional	2.0998	O25	O26
	2.4208	Zn5	Zn6		2.1028	O19	O20
	2.5392	O26	O28		2.1792	O23	O24
	2.6690	O22	O24		3.4614	O21	O22
	2.7072	Nb2	Nb4		3.5366	Nb2	Nb4

**Table 5:** Amount of adsorbed dye per unit mass of adsorbent and removal efficiency for a loading of 2 g/L.

	Methyl Red Concentration 1 × 10^−4^ M		Methyl Red Concentration 1 × 10^−5^ M	
Adsorbent Material	q_e_ [mg\g]	R.E. [%]	q_e_ [mg\g]	R.E. [%]
S-TE (control)	5.970	44.34	1.154	85.74
**S-TE-OX**	**11.550**	**85.78**	**1.191**	**88.42**
S-TE-porf	8.407	62.43	1.148	85.29
**S-TE-porf-OX**	**11.854**	**88.04**	**1.245**	**92.48**
S-TE-AP (control)	4.131	30.68	0.900	66.81
S-TE-AP-OX	1.529	11.36	0.814	60.45
S-TE-AP-porf	0.397	2.95	0.767	56.98
S-TE-AP-porf-OX	0.415	3.08	0.694	51.51

**Table 9:** Textural parameters of samples based on TEOS before exposure to MR.

Sample Name	Specific Surface Area [m^2^/g]	BJH Ads, Pore Size Distribution [nm]	BJH Des, Pore Size Distribution [nm]	Average Pore Size [nm]	Total Pore Volume [cm^3^/g]	FHH Desorption Branch
S-TE	726	3.068	3.732	3.064	0.556 for pores smaller than 171.5 nm	2.4779
S-TE-OX	569	3.477	3.701	2.912	0.415 for pores smaller than 169.9 nm	2.5452
S-TE-porf	674	3.465	3.721	3.114	0.525 for pores smaller than 259.5 nm	2.5112
S-TE-porf-OX	518	3.461	3.752	3.167	0.410 for pores smaller than 158.1 nm	2.4525

**Table 10:** Textural parameters of samples based on TEOS after exposure to MR.

Sample Name	Specific Surface Area [m^2^/g]	BJH Ads, Pore Size Distribution [nm]	BJH Des, Pore Size Distribution [nm]	Average Pore Size [nm]	Total Pore Volume [cm^3^/g]	FHH Desorption Branch
S-TE + MR	706	3.449	3.731	3.029	0.535 for pores smaller than 243.0 nm	2.5337
S-TE-OX + MR	563	3.459	3.740	3.341	0.470 for pores smaller than 157.3 nm	2.4410
S-TE-porf + MR	696	3.425	3.751	3.025	0.527 for pores smaller than 232.4 nm	2.5580
S-TE-porf-OX + MR	489	3.417	3.734	3.547	0.434 for pores smaller than 237.7 nm	2.4318

**Table 11:** Textural parameters of samples based on TEOS:APTMOS before exposure to MR.

Sample Name	Surface Area, [m^2^/g]	BJH Ads, Pore Size Distribution [nm]	BJH Des, Pore Size Distribution [nm]	Average Pore Size [nm]	Total Pore Volume [cm^3^/g]	FHH Desorption Branch
S-TE-AP	197	3.418	3.922	18.490	0.910 for pores smaller than 152.3 nm	1.4817
S-TE-AP-OX	199	3.452	3.378	13.033	0.649 for pores smaller than 151.7 nm	1.6392
S-TEAP-porf	145	3.432	3.930	11.150	0.403 for pores smaller than 141.6 nm	1.7750
S-TE-AP-porf-OX	131	3.453	3.227	12.219	0.401 for pores smaller than 147.8 nm	1.7584

**Table 12:** Textural parameters of samples based on TEOS:APTMOS after exposure to MR.

Sample Name	Surface Area, [m^2^/g]	BJH Ads, Pore Size Distribution [nm]	BJH Des, Pore Size Distribution [nm]	Average Pore Size [nm]	Total Pore Volume [cm^3^/g]	FHH Desorption Branch
S-TE-AP + MR	157	3.435	3.246	15.013	0.590 for pores smaller than 154.1 nm	1.5697
S-TE-AP-OX + MR	161	4.316	3.241	15.427	0.621 for pores smaller than 141.9 nm	1.5219
S-TE-AP-porf + MR	113	3.436	3.943	12.638	0.357 for pores smaller than 146.0 nm	1.6753
S-TE-AP-porf-OX + MR	118	3.838	3.940	12.701	0.374 for pores smaller than 143.0 nm	1.7132

**Table 13:** Sample abbreviations, silica precursors, catalysts, and incorporated materials.

No.	Silica Samples Abbreviation	Alcoxide Precursors (Molar Ratios)	HCl 37% (Moles)	TAPP Porphyrin (Moles)	Zn_3_Nb_2_O_8_ Doped with 0.5% Eu^3+^ (1% Reported to Alcoxide)	NH_3_ 2.5% (mol) Used Till Gelation
1	S-TE (control)	TEOS (1:1)-0.05 mole	0.001	-	-	0.00164
2	S-TE-OX			-	0.104 g	0.00203
3	S-TE-porf			5 × 10^−6^	-	0.00174
4	S-TE-porf-OX			5 × 10^−6^	0.104 g	0.00247
5	S-TE-AP (control)	TEOS/APTMOS (9:1)-0.045:0.005 mole	0.001	-	-	-
6	S-TE-AP-OX			-	0.104 g	-
7	S-TE-AP-porf			5 × 10^−6^	-	0.0025
8	S-TE-AP-porf-OX			5 × 10^−6^	0.104 g	0.0025

## Data Availability

The data presented in this study are available on request from the first author or the corresponding authors.

## Supplementary Materials

The supporting information can be downloaded at: https://www.mdpi.com/article/10.3390/ijms24108920/s1.
