# Supplementary material for: Nanomaterials Based on Collaboration with Multiple Partners: Zn3Nb2O8 Doped with Eu3+ and/or Amino Substituted Porphyrin Incorporated in Silica Matrices for the Discoloration of Methyl Red
Source: Int J Mol Sci. 2023 May 17;24(10):8920. doi: 10.3390/ijms24108920 (PMC10219493; doi:10.3390/ijms24108920)
Supplement: Supplementary file 1 [file ijms-24-08920-s001.zip › ijms-2362551-supplementary.pdf]

Nanomaterials based on collaboration of multiple partners: $Zn_3Nb_2O_8$ doped with Eu^{3+} and/or amino substituted porphyrin incorporated in silica matrices for the discoloration of methyl red

Mihaela Birdeanu [1,*], Ion Fratilescu [2], Camelia Epuran [2], Liviu Mocanu [1], Catalin Ianasi [2], Anca Lascu [2] and Eugenia Fagadar-Cosma [2,*]

[1] National Institute for Research and Development in Electrochemistry and Condensed Matter, Plautius Andronescu Street 1, 300224 Timisoara, Romania; mihaelabirdeanu@gmail.com (M.B.); mocanuliv@gmail.com (L.M.)
[2] Institute of Chemistry "Coriolan Dragulescu", Mihai Viteazu Ave. 24, 300223 Timisoara, Romania; ionfratilescu@acad-icht.tm.edu.ro (I.F.); ecamelia@acad-icht.tm.edu.ro (C.E.); alascu@acad-icht.tm.edu.ro (A.L.); ianasic@acad-icht.tm.edu.ro (C.I.); efagadar@yahoo.com (E.F.-C.)
* Correspondence: mihaelabirdeanu@gmail.com (M.B.) and efagadar@yahoo.com (E.F.-C.)

A comparison of the UV-Vis spectra in solid state for the two types of silica materials containing TAPP porphyrin, reveals that the silica materials starting from TEOS exhibit increased properties of absorption regarding the enlargement of the bands from 400 to 820 nm (important red shifting of the Q1 band of porphyrin) associated with significant hyperchromic effect. All these UV-Vis spectra (Figure S1) present the pattern of TAPP porphyrin, namely the broad Soret band associated with four Q bands of lower intensity.

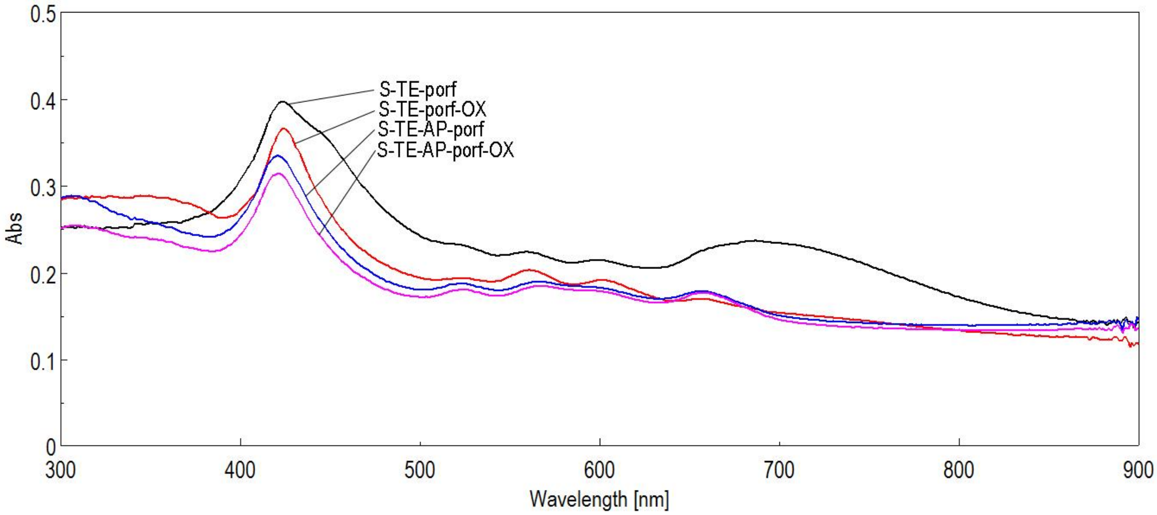

Figure S1. UV-Vis spectra in solid state for both types of silica materials containing porphyrin (TAPP).

Adsorption investigations using the hybrid silica materials as adsorbent with methyl red ($c = 1 \times 10^{-4}$ M) solution

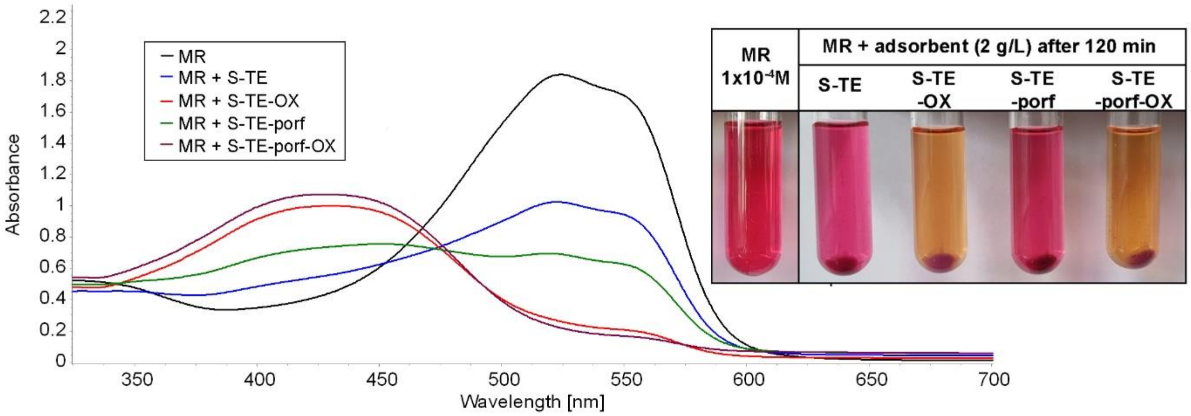

(**a**)

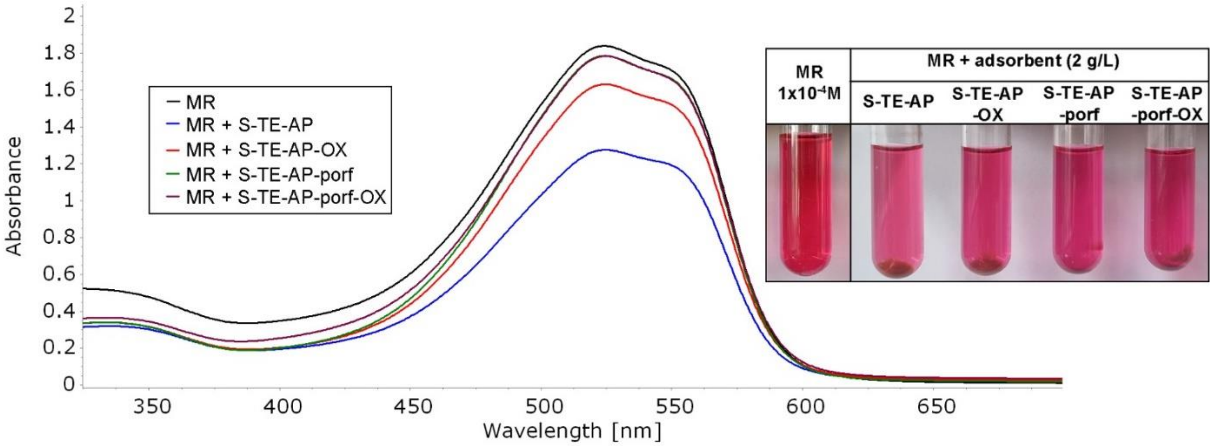

(**b**)

Figure S2. Overlapping UV-Vis spectra of the supernatant after 120 minutes exposure to a 5 mL MR solution ($c = 1 \times 10^{-4}$ M) for a loading of adsorbent material of **2g/L,** based on (**a**) TEOS and (**b**) TEOS:APTMOS precursors.

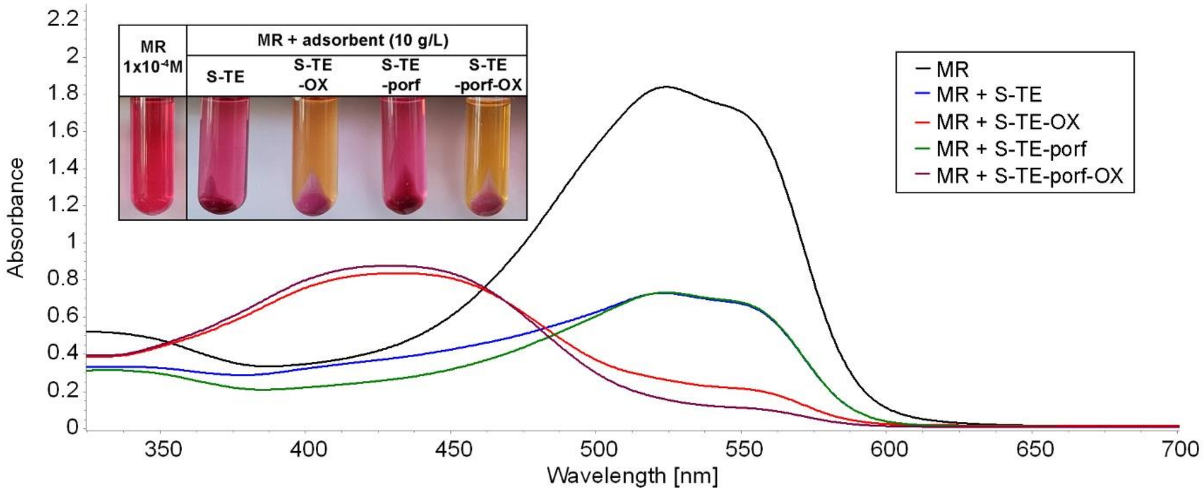

(**a**)

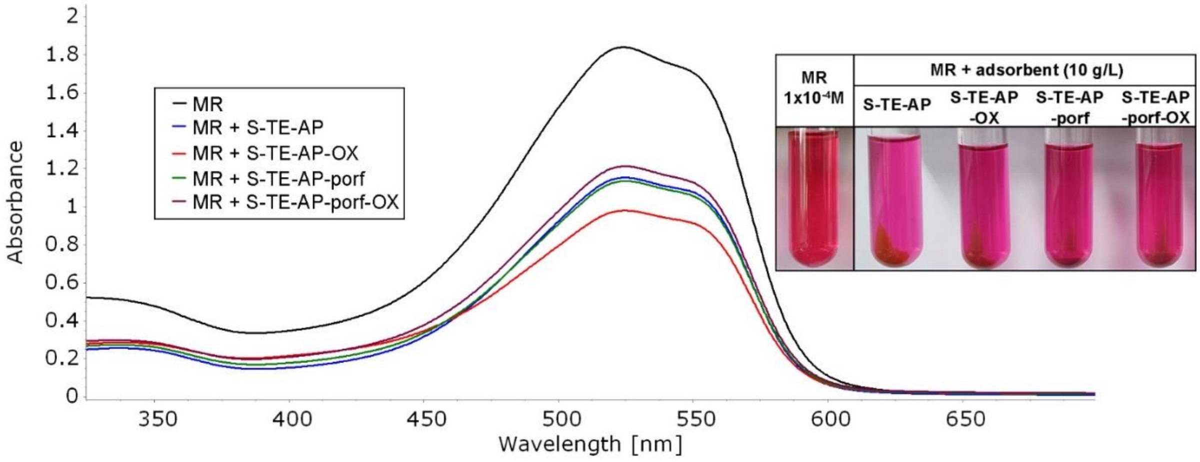

(**b**)

Figure S3. Superimposed UV-Vis spectra of the supernatant after 120 minutes exposure to a 5 mL MR solution (c = 1 × 10^{-4} M) for a loading of adsorbent material of **10 g/L,** based on (**a**) TEOS and (**b**) TEOS:APTMOS precursors.

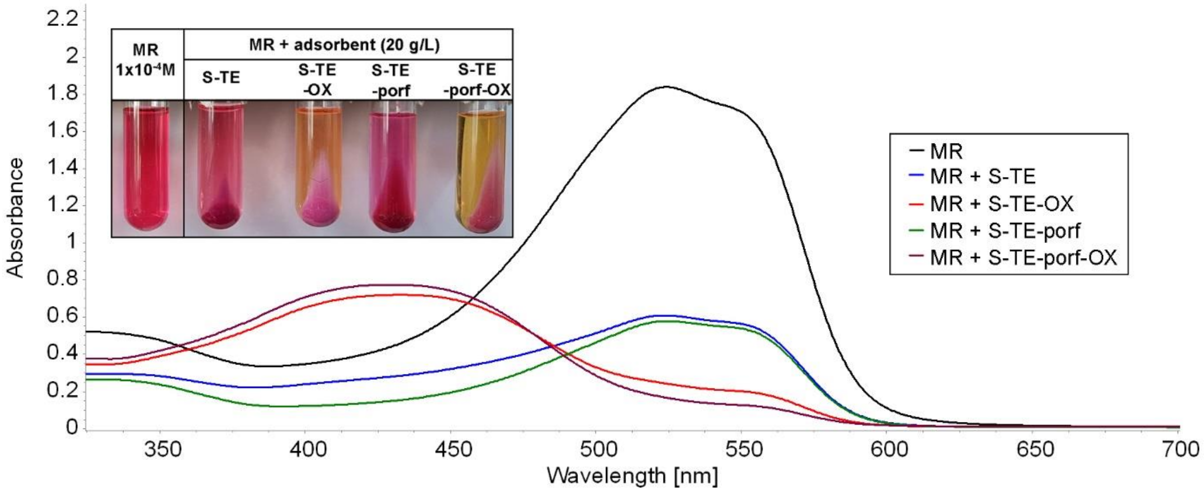

(**a**)

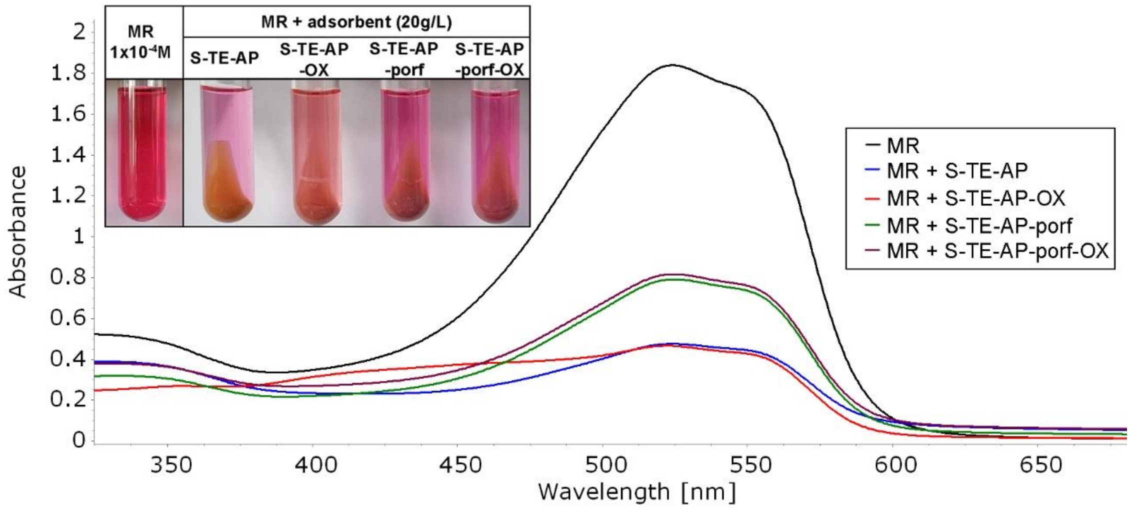

(**b**)

Figure S4. Superimposed UV-Vis spectra of the supernatant after 120 minutes of exposure to a 5 mL MR solution (c = 1 × 10^{-4} M) for a loading of adsorbent material of **20 g/L,** based on (**a**) TEOS and (**b**) TEOS:APTMOS precursors.
